# Limited codiversification of the gut microbiota within humans

**DOI:** 10.1128/mbio.03727-25

**Published:** 2026-07-06

**Authors:** Benjamin H. Good

**Affiliations:** 1 Department of Applied Physics, Stanford University 198871, Stanford, California, USA; 2 Department of Biology, Stanford University 118555, Stanford, California, USA; 3 Biohub, San Francisco, California, USA; University of California, Irvine, Irvine, California, USA

**Keywords:** human gut microbiome, population genetics, phylogeography

## Abstract

Gut bacteria exhibit striking variation across different human populations, but the evolutionary forces that have shaped this diversity are less well understood. Recent work has argued that many species of gut bacteria have codiversified with modern humans, based on the phylogenetic correlations between human and microbial genomes. Here, I re-analyze these data and show that the correlations between human and microbial phylogenies are often substantially weaker than those between unlinked human chromosomes and that similar correlations can arise through geographic structure alone. These results suggest that traditional codiversification has been limited in recent human history and highlight alternative strategies for quantifying the extent of human-microbe coevolution.

**IMPORTANCE:**

There is widespread interest in understanding the evolutionary history of our gut microbiota and how it varies within and among different human population groups. This Observation critically re-examines the hypothesis that many commensal gut bacteria have evolved in parallel (or “codiversified”) with modern humans, providing new evidence that the correlations between human and microbial genealogies are weaker than previously supposed. These findings have important evolutionary implications and also practical consequences, from the sourcing of probiotic therapies to the design of sequencing-based diagnostics.

## OBSERVATION

The human microbiome is highly diverse, with individuals varying in both the species they contain as well as the genetic variants (or “strains”) of each species (1). Advances in metagenomic sequencing have made it possible to catalog this intra-species variation in human populations from around the world (2–8). Understanding the origins and implications of this diversity—and how it depends on the lifestyle and ancestry of the host—is an important frontier of microbiome research.

A recent study by Suzuki et al. (8) shed new light on this problem by assembling a large collection of sequenced human gut metagenomes and paired host genomes from several industrialized populations around the world. The authors used these data to argue that dozens of species of gut bacteria have evolved in parallel (or “codiversified”) with different human population groups, based on the observed genetic correlations between their human and microbial genomes (8). An accompanying perspective (9) proposed a stronger model, in which gut bacteria may have kept fidelity to human lineages for thousands of human generations. Such intimate commensal relationships would be remarkable if true, since they would create extensive opportunities for coevolution between human and bacterial genomes.

While striking examples of codiversification have been observed between host species (10–12), they are more challenging to detect on the shorter evolutionary timescales separating modern human populations. The underlying genetic distance scales of humans and commensal gut bacteria are roughly compatible with each other. Estimates of the mutation accumulation rate (13, 14) suggest that pairs of isolated bacterial lineages should have diverged by ∼1%–10% in the ∼60,000 years since the out-of-Africa expansion (see the supplemental methods). These divergence levels are comparable to the observed synonymous diversity within many species of human gut bacteria (15), potentially hinting at a common evolutionary origin.

However, similar to our own genomes, the genetic diversity of the gut microbiota is highly intermingled across space and time. Previous work has shown that the divergence between conspecific strains from different continents is often comparable to the diversity among strains from the same geographic location (2, 7, 15). Horizontal transmission is also widespread. Unrelated hosts from different continents can occasionally share nearly identical bacterial strains (15–17), while even the most closely related hosts—identical twins—are often colonized by more distantly related strains over their lifetimes (15).

To identify signatures of intra-specific codiversification against this backdrop, Suzuki et al. (8) compared phylogenetic trees reconstructed from single nucleotide variants within their human and microbial genomes (see the supplemental methods). This is a well-established approach in host-symbiont coevolution (10). Yet some care must be taken when interpreting genome-wide phylogenies on shorter within-species timescales. In addition to natural selection (15, 18), frequent homologous recombination within both humans and gut bacteria (15, 17) produces mosaic patterns of ancestry that are rarely captured by a single phylogenetic tree. Despite these caveats, recent work has shown that genome-wide phylogenies inferred from rapidly recombining populations can still encode some information about the historical patterns of gene flow within a species (19). In such settings, any observed similarities between the human and bacterial phylogenies will tend to reflect the cumulative effects of gene flow among larger ancestral populations (20), rather than the genealogical fidelity of individual microbial lineages.

**Fig 1:**
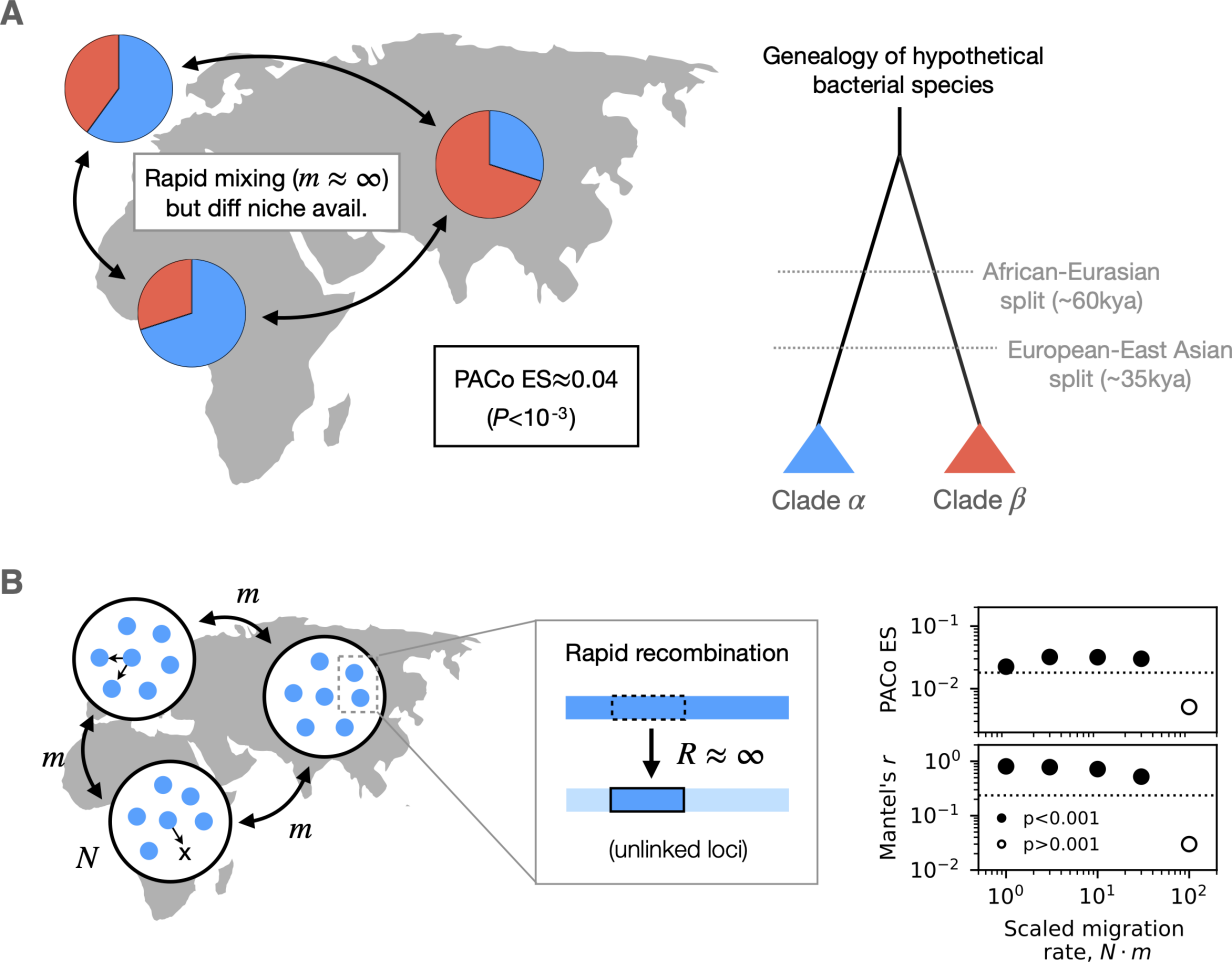
Geographic structure can produce statistically significant phylogenetic correlations even in the absence of codiversification. (**A**) An example of a hypothetical bacterial species that did not evolve in parallel with modern humans. Two genetically isolated clades originated well before the out-of-Africa migration (right) and are both currently shared among all human populations. Subtle biases in the relative frequencies of the clades across continents (unrelated to human ancestry) can produce spurious correlations with the observed human phylogeny (left) that are comparable to the largest effect sizes observed by Suzuki et al. (8); PACo scores for this example were calculated using the observed human phylogeny for the subset of hosts in Suzuki et al. (8) that contained strains of *Prevotella copri*. (**B**) An alternative geographic null model with finite rates of migration and widespread within-species recombination. In this example, bacterial recombination occurs infinitely quickly within each continent, leading to rapid unlinking of mutations. Genome-wide phylogenies inferred from simulations of this model exhibit significant correlations with the observed human phylogeny (see the supplemental methods), even in regimes where most local strains have recently migrated from another continent ($mT_{mrca}≳Nm\gg 1$). Points denote simulation results and their associated *P*-values, while the dashed lines show the corresponding levels observed for *P. copri* (see the supplemental methods). These two theoretical examples show that the presence of phylogenetic correlations between two species does not necessarily imply that they followed a parallel evolutionary history.

To quantify these signals, Suzuki et al. (8) used a phylogenetic congruence test (PACo) to measure the similarities between pairs of human and bacterial phylogenies. By permuting the links between hosts and bacteria, they found that 36 of 59 species were more similar to the human phylogeny than expected by chance (*q* < 0.05). While these significant *q*-values show that the human and bacterial phylogenies are not completely independent of each other, they do not directly demonstrate the evolutionary parallelism implied by traditional models of codiversification (21). For example, theoretical arguments show that small amounts of geographic structure can also produce statistically significant PACo scores even in the absence of a shared evolutionary history (Fig. 1), since human genetic diversity is already separately correlated with geography (22). The presence of such confounders suggests that additional information is needed to interpret the evolutionary mechanisms driving the significant PACo scores in studies like Suzuki et al. (8).

**Fig 2:**
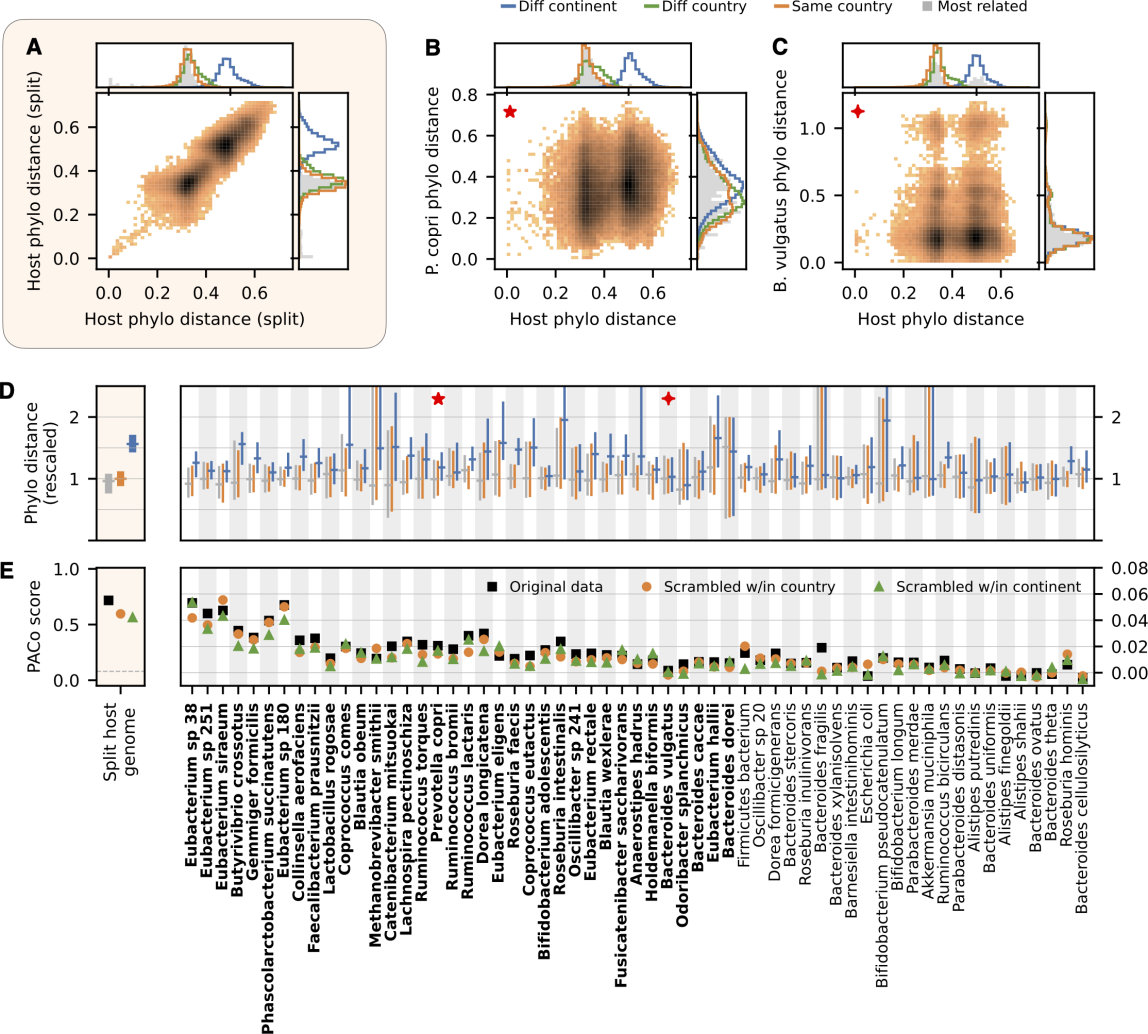
Host genetic similarity is not strongly predictive of bacterial genetic similarity. (**A–C**) Phylogenetic correlations for separate halves of the human genome (**A**), *Prevotella copri* (**B**), and *Bacteroides vulgatus* (**C**). Heat map shows estimated phylogenetic distances between pairs of adult individuals sequenced by Suzuki et al. (8); three samples with anomalously large host distances (Fig. S4) were removed so that they would not bias the correlation in panel A. Right histogram panel shows the distribution of bacterial distances for hosts in different geographic categories (blue, orange, and green), or in the next most closely related hosts (gray); top histogram panel shows analogous distributions for host phylogenetic distance, using the next most closely related bacterial strain to construct the gray distribution. (**D**) Summarized versions of the right histograms in panels A–C for all of the species analyzed by Suzuki et al. (8). Colored lines show medians and interquartile ranges, rescaled by the median within-country distance for each species. (**E**) PACo effect sizes for all of the species shown in panel D. Note that the left and right sub-panels have different scales to aid in visualization. Squares denote the recomputed effect sizes for the original data, while circles and triangles show analogous values obtained when the host labels are separately permuted across hosts from the same country (circles) or continent (triangles). Species with statistically significant PACo scores (*q* < 0.05) in Suzuki et al. (8) are indicated in bold.

In principle, unlinked regions of the human genome could provide a positive control for sequences that have truly codiversified with each other, while relaxing the stronger assumption of strict genealogical fidelity. Unlike mitochondria, which are strictly maternally inherited, different halves of the nuclear genome autosomes trace their ancestry back to hundreds of distinct genetic ancestors within a few hundred years, similar to a large random sample from the same local population (23). From an evolutionary perspective, this rapid decoupling of genetic ancestry resembles a form of effective “horizontal transmission” within the local population of potential mates. The residual correlations between these unlinked chromosomes could therefore provide a natural null model for codiversification that does not rely on strict maternal inheritance, as previously observed in extreme cases of endosymbiosis (10).

Interestingly, repeating the same PACo analysis on separate halves of the human genome yields a PACo effect size of ES ≈ 0.7 (Fig. 2), substantially greater than the largest microbial effect sizes reported by Suzuki et al. (8) (ES ≲ 0.07). Similar values are obtained when the host labels are separately randomized among individuals from the same country or continent (Fig. 2E), demonstrating that they are driven by phylogenetic correlations at the largest geographic scales. This large gap in effect sizes suggests that the observed correlations between the human and bacterial phylogenies are substantially lower than expected under more relaxed models of codiversification that allow for local “horizontal” transmission.

These results are not specific to the PACo test. Direct examination of the phylogenetic distances reveals that two halves of the human genome are substantially more correlated with each other than with any gut bacterial genomes (Fig. 2A through C; Fig. S1). For species with the strongest reported codiversification scores (e.g., *Prevotella copri*; Fig. 2B), one can observe a small but statistically significant decrease in bacterial relatedness with increasing host distance (*P* < 10^−3^, Mantel test; Fig. S2). However, these small average differences are dwarfed by the strain variation within individual countries (Fig. 2B, right, orange) or among the most closely related hosts (gray). A similar behavior can be observed in other species with significant PACo scores (e.g., *Bacteroides vulgatus*; Fig. 2C), though the subtle shifts in mean relatedness across continents can be more difficult to discern by eye (*P* < 10^−3^, Kolmogorov-Smirnov test). In both cases, even the most closely related hosts contain bacteria that span nearly the full range of bacterial relatedness, while hosts from different continents frequently share strains that are as similar as those from the most closely related hosts (Fig. 2D; Fig. S3 and S4). These findings are consistent with previous observations in adult twins (15), suggesting that they do not sensitively depend on the phylogenetic inference scheme employed here (Fig. S5).

Together, these results suggest that traditional codiversification has been limited within modern human populations. Gut bacteria may have still adapted to specific groups or geographic locales. But for most of the species and populations examined here, the genome-wide diversity of their constituent strains does not strongly mirror the evolutionary history of their host populations.

This distinction has important evolutionary implications, as well as potential practical consequences. Strong forms of codiversification might imply that it would be beneficial to source probiotic therapies or other microbiome treatments based on the geographic origin or genetic ancestry of the host. In contrast, the large range of phylogenetic distances in Fig. 2 suggests that such therapies might be better informed by directly genotyping the microbiota (rather than the host), or by identifying smaller sub-regions of microbial genomes that exhibit stronger geographic associations (24). In the latter case, lower genome-wide levels of codiversification could make it easier to detect such locally adapted regions, by searching for parallel genes or haplotypes across otherwise phylogenetically distinct genomic backgrounds (17, 25). Stronger signals of codiversification might also be present in human populations with particularly strong and/or ancient geographic separation (7), which could reduce the rates of microbial gene flow at the largest geographic scales. Further efforts to explore these possibilities represent promising avenues for future work.

In light of the above results, it is interesting to ask why the bacterial phenotypes that were most associated with stronger phylogenetic correlations (e.g., genome size or poor growth at 27°C [8]) have not led to larger genetic differences in certain species, like *P. copri*, given their dramatically reduced ability to survive outside their hosts. The results above suggest that subtle differences in the rates of gene flow and recombination could play a crucial role in shaping these large-scale geographic patterns, even if they are not directly related to the historical process of codiversification. Understanding the ecological and evolutionary mechanisms that allow commensal gut bacteria to spread between hosts—and the signatures this leaves in their genomes—will be critical for furthering our understanding of how humans and their microbiota have coevolved.

## Data Availability

Phylogenies and alignments from Suzuki et al. (8) were downloaded from the Dryad repository provided in that work; host and bacterial metadata were obtained from the Supplementary Materials of that work. All analysis code used in this work is available on GitHub (https://github.com/bgoodlab/codiversification_microbiome).

## SUPPLEMENTAL MATERIAL

Supplemental Material mbio.03727-25-s0001.pdf Supplemental methods and Fig. S1 to S5.

ASM does not own the copyrights to Supplemental Material that may be linked to, or accessed through, an article. The authors have granted ASM a non-exclusive, world-wide license to publish the Supplemental Material files. Please contact the corresponding author directly for reuse.
