# Supplementary material for: Limited codiversification of the gut microbiota within humans
Source: mBio. 2026 Jul 6;17(8):e03727-25. doi: 10.1128/mbio.03727-25 (PMC13463816; doi:10.1128/mbio.03727-25)
Supplement: Supplementary file 1 [file mbio.03727-25-s0001.pdf]

Supplemental Methods

Source Data. Phylogenetic trees and alignments from Ref. (1) were downloaded from the associated Dryad repository (2). The microbial phylogenies in this study were inferred using StrainPhlAn v3.0 (3), which considers patterns of single nucleotide variation within a panel of species-specific marker genes. The corresponding human genotypes were inferred for a panel of known single nucleotide polymorphisms using an Illumina genotyping array (1). Manual examination of the human genotype data revealed that three Cameroonian subjects were highly diverged from other Cameroonians and from the rest of the study cohort (Fig. S4). Since the genomes of their gut bacteria were not similarly diverged from the rest of the sample, we removed these three individuals from all analyses (except for Fig. S4) so that they would not inflate the relative correlations between different halves of the human genome.

Phylogenetic correlations within the human genome. Comparisons between different halves of the human genome were performed by splitting the human genotype alignments into two equal subsets. Phylogenies were re-inferred for each set using SNPhylo (4), following the same procedures and parameters described in Ref. (1). Phylogenetic distances were extracted form the corresponding phylogenies, while raw genetic divergences were computed as the fraction of mismatches within the subset of alignable sites for each pair (Fig. S5). PACo scores were computed using a modified version of the code provided by Ref. (1).

Phylogenetic correlations in simple models of geographic structure. To demonstrate that phylogetic correlations can arise even in the absence of codiversification, we considered two hypothetical models of non-codiversifying bacteria that are illustrated in Fig. 1.

In the first model, bacterial genomes were sampled from the phylogeny in Fig. 1A: genomes from the same clade had a star-like phylogeny, with an internal phylogenetic distance of $d_i \approx 0.3$, while branch lengths separating the two clades were set to $d_o \approx 1$. For each of the 521 subjects in the original dataset that possessed a *Prevotella copri* strain, we randomly sampled a hypothetical microbial genome from one of the two clades in the relative proportions illustrated in Fig. 1A ($p_\alpha \approx 0.7$, 0.6, and 0.3, for Africa, Europe, and Asia respectively). These proportions were purposefully chosen to have a different topology than the genetic relatedness between the three human populations, to emphasize that they are independent of human ancestry. This hypothetical microbial phylogeny was then combined with the observed human phylogeny to calculate an equivalent PACo score, using the same methods described above.

In the second model, we simulated the opposite extreme of a rapidly recombining population with finite rates of geographic mixing. We considered a standard neutral island model with three equal-sized populations representing the three different continents in Fig. 1. Each continent was assumed to be perfectly well-mixed, while migration between continents occurred at a uniform per capita rate m. Recombination was assumed to occur sufficiently rapidly within each continent that the mutations at each locus could be sampled independently. This allowed us to simulate the model in a more efficient manner by performing a large number of single-locus simulations across a range of population-scaled migration rates (Nm). We assumed an infinite sites limit, in which each sampled polymorphism was founded by a single mutation event. For each value of Nm, a sample of hypothetical microbial genomes was constructed by sampling $L = 2 \times 10^4$ independent alleles from the simulated matrix of allele frequencies across continents. The number of polymorphisms (L) was chosen to coincide with the typical genetic diversity of human gut bacteria (5).

From these simulated microbial genome alignments, we inferred a genome-wide phylogeny using RAxML (6) using the same procedures described in Ref. (1). We then combined this simulated phylogeny with the observed human phylogeny to estimate PACo scores in Fig. 1B. We also performed a standard Mantel test (7) using the matrix of phylogenic distances. For both metrics, we observed statistically significant phylogenetic correlations with the human genome even under high scaled migration rates ($Nm \gg 1$). In this limit, most sampled genomes will have recently migrated from another continent, on a timescale much shorter than the typical TMRCA ($\propto N$).

Estimates of the bacterial molecular clock. To estimate the bacterial genetic divergence accumulated since the out-of-Africa expansion, we used two different estimates of the bacterial molecular clock. Direct observations in *E. coli* (8) and *B. fragilis* (9) are consistent with *in vivo* substitution rates of $r \sim 10^{-7} - 10^{-6}$ per bp per year. Compounded over the estimated ∼60,000 years since the out-of-Africa expansion (10), this leads to an estimated divergence of

$$d = 2 \cdot \left(10^{-7} - 10^{-6}\right) \cdot 6 \times 10^{4} \approx 1 - 10\%. \tag{1}$$

at neutral loci (e.g. synonymous sites) between isolated pairs of strains. Similar (though slightly cruder) bounds can also be estimated from first principles using mutation rates measured in *in vitro* mutation accumulation experiments ($\sim 10^{-9} - 10^{-10}$ / bp / gen; 11). Given an estimated growth rate of 1-10 generations per day (12), this leads to a an estimated substitution rate of $r \sim 10^{-7.5} - 10^{-5.5}$ per bp per year, which increases the range of d by a factor of 3 in each direction. We note that these estimates should be treated cautiously, given the considerable uncertainty in the underlying mutation rates and generation times that gut bacteria experience *in vivo*. Nevertheless, this order-of-magnitude precision is sufficient to show that the timescales of human population history will generally fall within the scale of a single bacterial species, rather than the longer evolutionary timescales that are traditionally associated with host-microbe codiversification (13).

Code availability. All analysis code used for data processing and figure generation is available on Github (`https://github.com/bgoodlab/codiversification_microbiome`).

Supplemental References

[1] T. A. Suzuki, *et al.*, *Science* **377**, 1328 (2022).

[2] T. Suzuki, L. Fitzstevens, N. Youngblut, R. Ley, *Dryad, Dataset* p. https://doi.org/10.5061/dryad.qrfj6q5k2 (2022).

[3] D. T. Truong, A. Tett, E. Pasolli, C. Huttenhower, N. Segata, *Genome Research* **27**, 626 (2017).

[4] T.-H. Lee, H. Guo, X. Wang, C. Kim, A. H. Paterson, *BMC genomics* **15**, 1 (2014).

[5] N. R. Garud, B. H. Good, O. Hallatschek, K. S. Pollard, *PLoS Biology* **17**, e3000102 (2019).

[6] A. Stamatakis, *Bioinformatics* **30**, 1312 (2014).

[7] K. Hommola, J. E. Smith, Y. Qiu, W. R. Gilks, *Molecular biology and evolution* **26**, 1457 (2009).

[8] M. Ghalayini, *et al.*, *Appl. Environ. Microbiol.* **84**, e02377 (2018).

[9] S. Zhao, *et al.*, *Cell Host & Microbe* **25**, 656 (2019).

[10] A. Bergström, C. Stringer, M. Hajdinjak, E. M. Scerri, P. Skoglund, *Nature* **590**, 229 (2021).

[11] W. Sung, M. S. Ackerman, S. F. Miller, T. G. Doak, M. Lynch, *Proc Natl Acad Sci USA* **109**, 18488 (2012).

[12] O. M. Ghosh, B. H. Good, *Proc Natl Acad Sci USA* **119**, e2114931119 (2022).

[13] N. A. Moran, J. P. McCutcheon, A. Nakabachi, *Annual Review of Genetics* **42**, 165 (2008).

Supplemental Figures

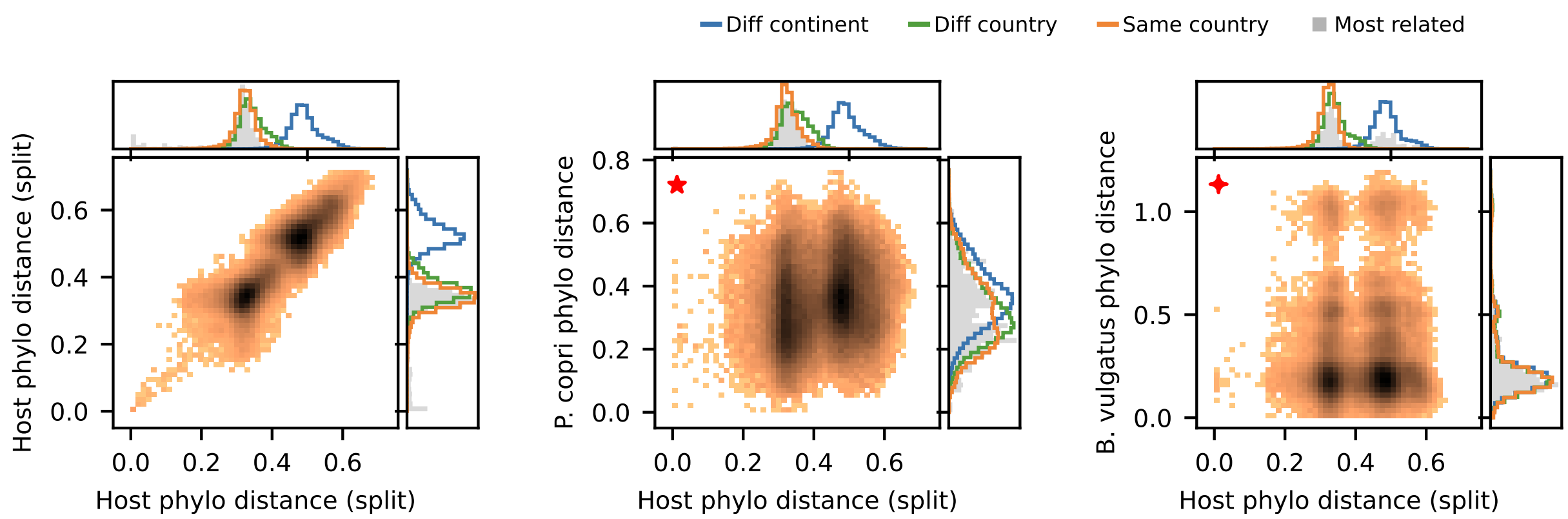

Figure S1: Analogous versions of Fig. 2B,C using the same x axis as panel A.

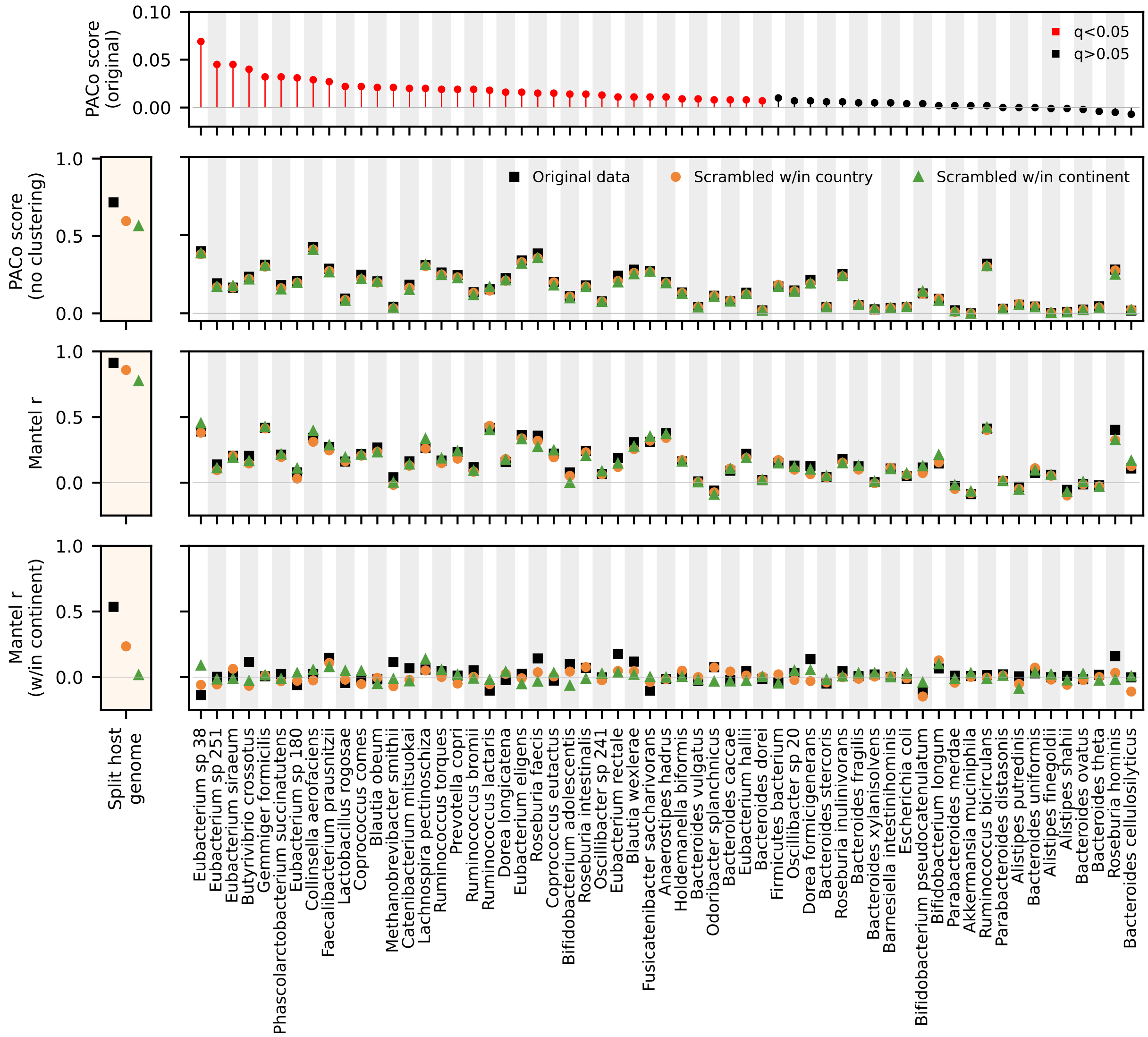

Figure S2: Analogous versions of Fig. 2E for other phylogenetic correlation metrics. Top panel shows the original PACo effect sizes and q-values computed by Suzuki & Fitzstevens *et al* (1). These differ slightly from the values shown in Fig. 2E due to the finite number of bootstrapped samples ($n \approx 1000$). The second panel shows an analogous set of PACo scores computed without the hierarchical clustering step in Ref. (1), which was applied to the microbial phylogenies but not their human counterparts. The third panel shows the corresponding effect sizes for a Mantel test (7), which measures the correlations between the pairwise distances in the human and microbial phylogenies. The forth panel shows an analogous set of Mantel tests restricted to pairs of intra-continental comparisons. These data show that the gap between the intra-human and human-microbe comparisons is preserved across other phylogenetic correlation metrics.

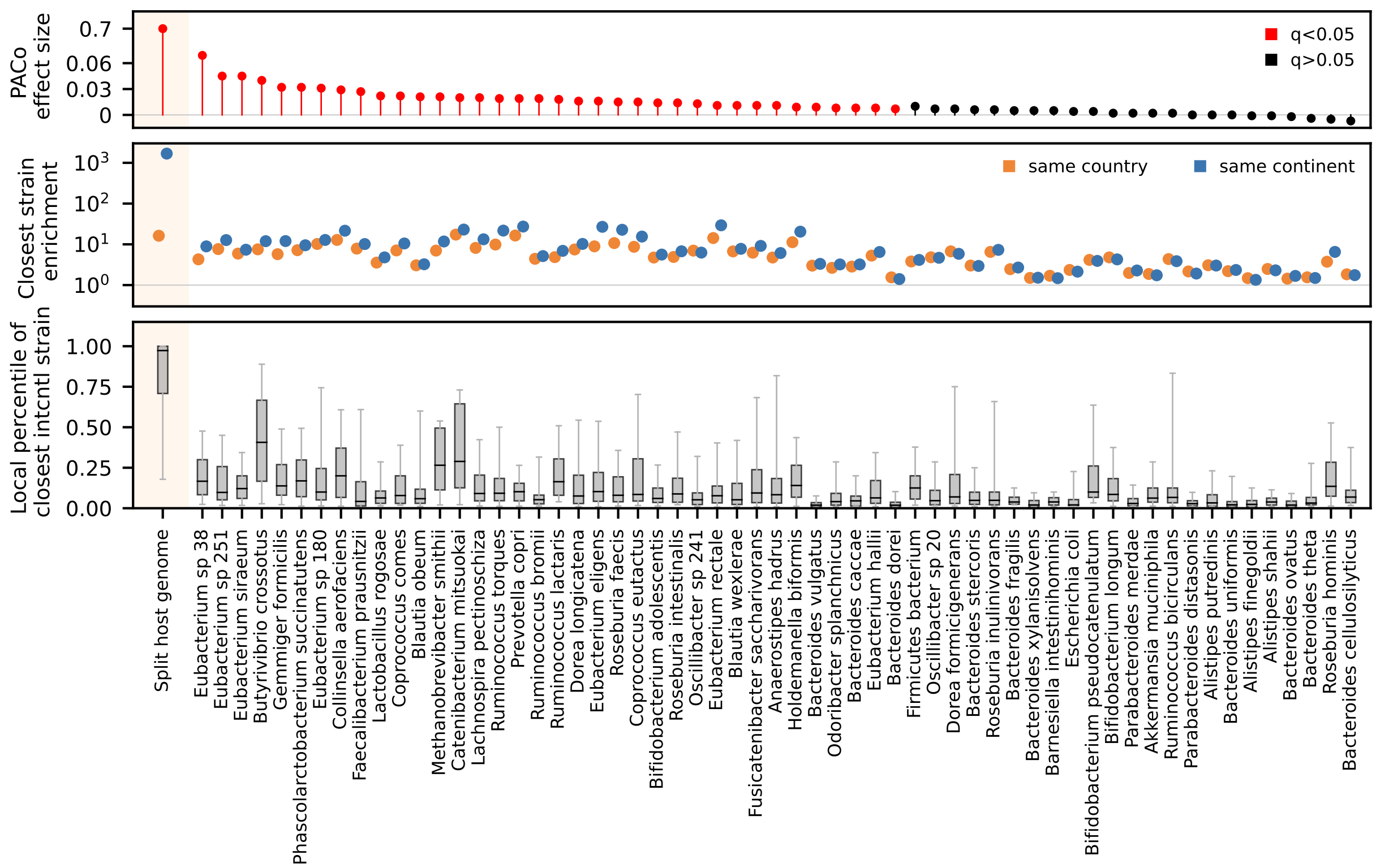

Figure S3: Closely related bacterial strains within and across continents. Top panel shows the same PACo effect sizes in Fig. S2 (top). Middle panel shows the enrichment of the probability that the closest relative of a strain derives from the same country (orange) or continent (blue). The enrichment factor is calculated as $e = f(1-f_0)/(1-f)f_0$, where f is observed fraction of strains whose closest relative is located in the same country (or continent) and f_0 is the expected fraction if the strains were randomly permuted across countries. These data show that closest bacterial strains are more likely to come from the same country or continent, but less frequently than their human hosts. Bottom: the phylogenetic distance percentile of the closest inter-continental strain, relative to the set of strains from the same country. These data show that most strains have close relatives on other continents (e.g. in the top quartile of the within-country distribution).

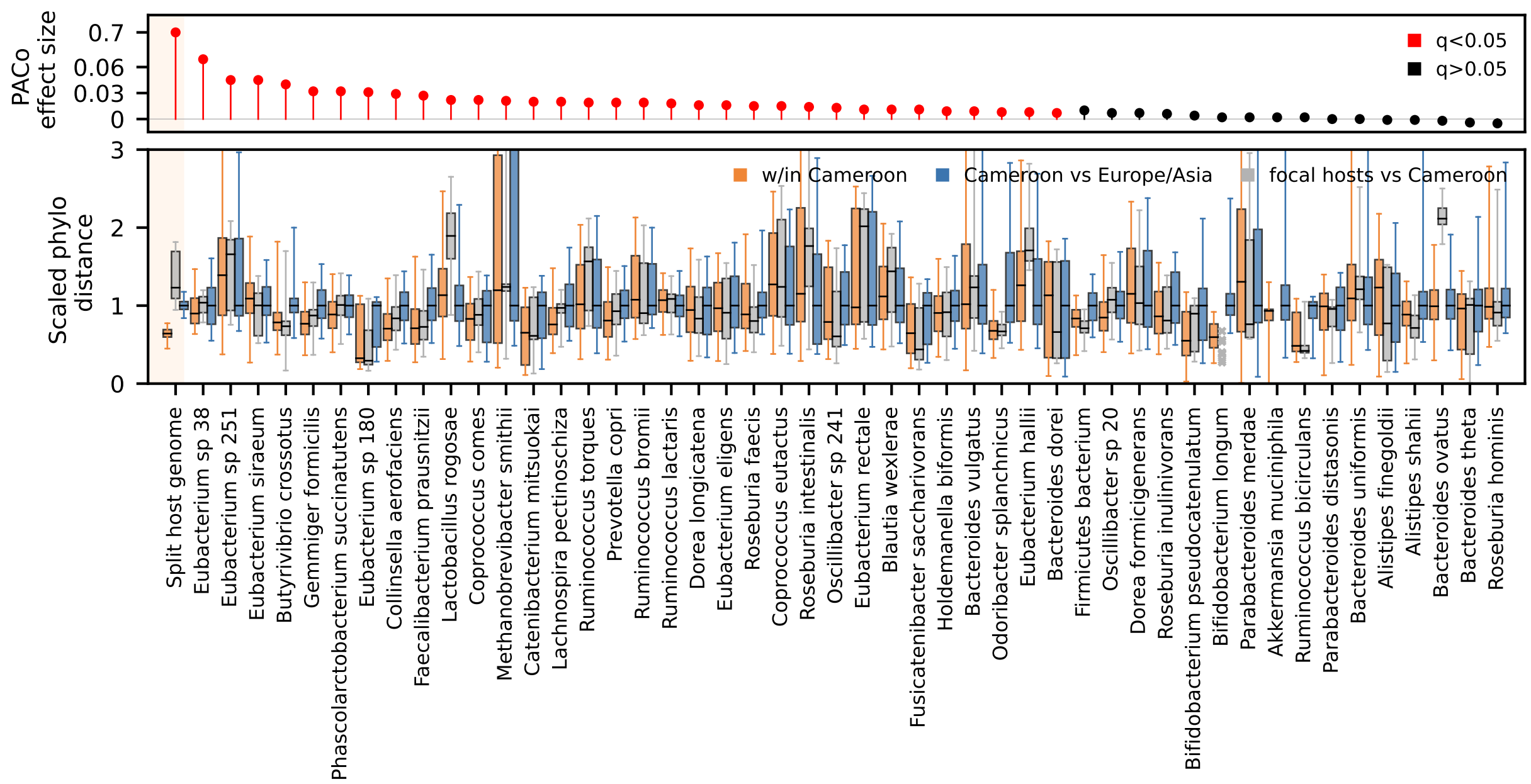

Figure S4: Limited signatures of codiversification in the most highly diverged hosts. Three hosts from Cameroon (BEB30, BEN18, BEN43) were highly diverged from other Cameroonians and from the rest of the human cohort (bottom, left). The bottom panel shows that for most of the species considered, the divergence of the strains from these three genetically diverged hosts relative to other Cameroonians comparable to the local strain diversity within Cameroon, independent of the species' PACo effect size (top). To aid visualization, the phylogenetic distances in the bottom panel are scaled by the median inter-continental distance within each species.

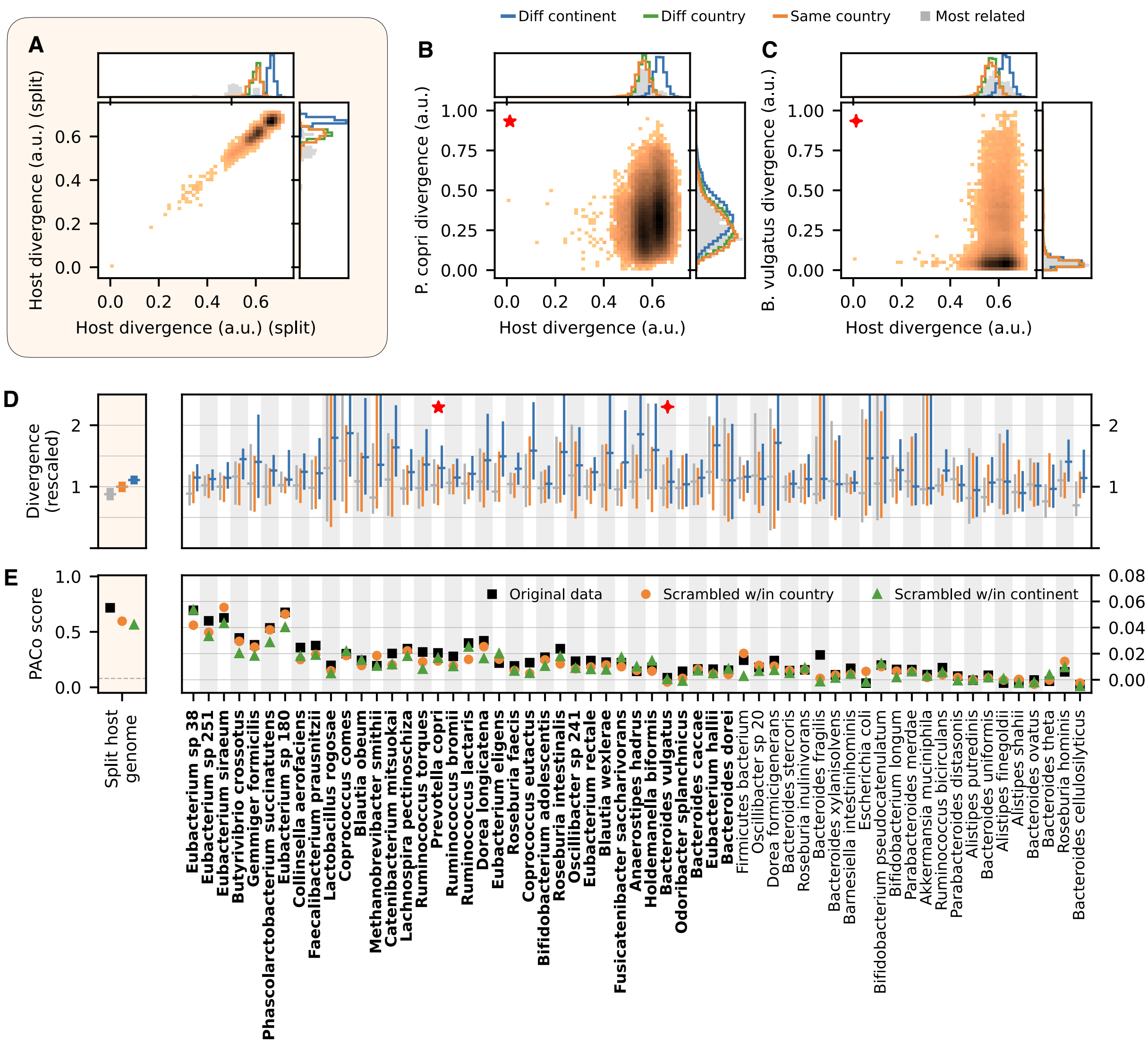

Figure S5: Analogous versions of Fig. 2A-D computed using raw genetic divergences rather than the inferred phylogenetic distances. The PACo scores are the same as those shown in Fig. 2E.
